# Epidural Analgesia with Ropivacaine during Labour in a Patient with a SCN5A Gene Mutation

**DOI:** 10.1155/2016/9278409

**Published:** 2016-09-07

**Authors:** A. L. M. J. van der Knijff-van Dortmont, M. Dirckx, J. J. Duvekot, J. W. Roos-Hesselink, A. Gonzalez Candel, C. D. van der Marel, G. P. Scoones, V. F. R. Adriaens, I. J. J. Dons-Sinke

**Affiliations:** ^1^Department of Anesthesiology, Erasmus University Medical Centre, Rotterdam, Netherlands; ^2^Department of Obstetrics and Gynaecology, Erasmus University Medical Centre, Rotterdam, Netherlands; ^3^Department of Cardiology, Erasmus University Medical Centre, Rotterdam, Netherlands

## Abstract

SCN5A gene mutations can lead to ion channel defects which can cause cardiac conduction disturbances. In the presence of specific ECG characteristics, this mutation is called Brugada syndrome. Many drugs are associated with adverse events, making anesthesia in patients with SCN5A gene mutations or Brugada syndrome challenging. In this case report, we describe a pregnant patient with this mutation who received epidural analgesia using low dose ropivacaine and sufentanil during labour.

## 1. Introduction

There are several known SCN5A gene mutations, leading to ion channel defects causing either decreased sodium or calcium influx or increased potassium efflux from the cardiomyocyte [1]. Patients may suffer syncope or sudden cardiac death secondary to polymorphic ventricular tachycardia or ventricular fibrillation. However, the majority of patients remain completely asymptomatic. A small percentage of patients with this gene mutation suffer from Brugada syndrome (BrS). This is a rare, autosomal dominant, arrhythmogenic disorder characterized by the presence of a typical electrocardiographic (ECG) pattern (right bundle branch block and persistent ST-segment elevation in the right precordial leads) and it is associated with a risk of sudden cardiac death [2]. The only effective prevention of sudden cardiac death in patients with heart rhythm disturbances is an implantable cardioverter defibrillator (ICD) [3].

Many drugs have been associated with arrhythmogenic events in BrS patients. Because of the unknown phenotype in patients with a SCN5A gene mutation, avoidance of arrhythmogenic medication is advised [3]. Bupivacaine is one of the drugs that should be avoided (evidence class II a) [4]. Spinal anesthesia using bupivacaine has been used in pregnant [5] and male patients without provoking arrhythmias [6, 7]. A pregnant, SCN5A-positive patient had an uneventful epidural during labour and delivery using a 10 mL bolus of bupivacaine 0,125% followed by an epidural infusion of bupivacaine 0,125% plus 2 *μ*g fentanyl at 10 mL/hour, without any recorded ECG changes during labour [8].

In this case report, we describe the use of low dose ropivacaine for epidural analgesia during labour and delivery in a patient with a SCN5A mutation.

## 2. Case Report

**Figure 1:**
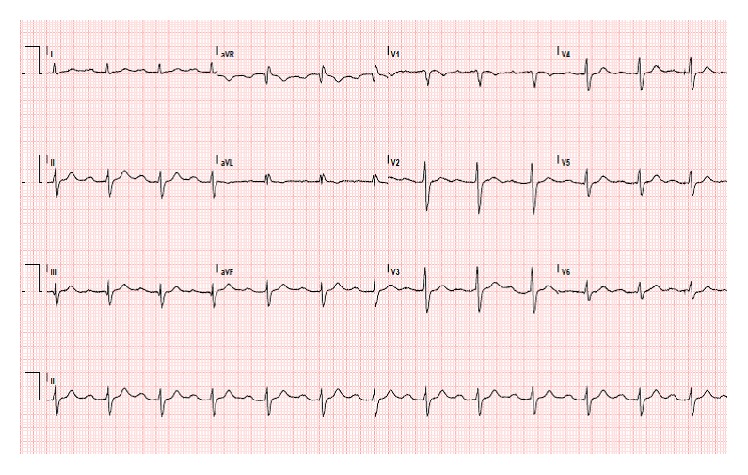
Patient's ECG, showing a first degree heart block with an intraventricular conduction delay. PR interval: 302 ms, QRS duration: 144 ms, and ventricular rate: 76 beats per minute.

A 31-year-old patient, carrier of SCN5A mutation, with no history of syncope or aborted sudden cardiac arrest, presented at the preoperative assessment clinic of our anesthetic department at 27 weeks of gestation. She used no medication. Past medical history included surgical correction of an atrial ventricular septum defect at the age of 8. Cardiac ultrasound showed mild left sided atrioventricular valve regurgitation and a good left ventricular function. An ECG showed a first degree heart block with an intraventricular conduction delay; see Figure 1. Because of these findings, she was included in a clinical research project relating to the association of cardiac septum defects, conduction disturbances, and SCN5A mutations. Her genotype showed a splice-site mutation c.4719C>T in exon 27 of the SCN5A gene. This rare mutation, which was only once found in another patient in Netherlands, is the most likely cause of her cardiac conduction disturbances. The father of our patient who also has cardiac conduction disturbances was found to have the same gene mutation.

The obstetric history included one spontaneous abortion and one instrumental vaginal delivery of a healthy male neonate complicated by a retained placenta requiring manual removal under uncomplicated general anesthesia. With regard to the SCN5A mutation and local anesthetics at her first labour, epidural analgesia was considered a relative contraindication due to the possibility of arrhythmias and she was offered patient-controlled analgesia using remifentanil.

Having experienced her previous delivery as being very traumatic, she specifically requested epidural analgesia during labour in her current pregnancy. After a multidisciplinary discussion with our cardiologists, gynaecologists, and anesthesiologists, it was agreed to induce labour at 38 weeks of gestation with early titrated epidural analgesia under continuous rhythm monitoring. Ropivacaine 0.1% with sufentanil 1 *μ*g/mL was considered the safest anesthetic medication. The patient was fully informed about the risks and possible adverse events.

At 38 weeks of pregnancy, she was admitted at the department of obstetrics. Monitoring consisted of continuous ECG, saturation, and noninvasive blood pressure monitoring and the presence of a resident cardiologist.

The epidural space was located at 5 cm using 18-gauge Tuohy needle at the L3-4 intervertebral space. An epidural catheter was inserted and a test dose of 2.5 mL ropivacaine 0.1% with sufentanil 1 *μ*g/mL was administered resulting in no changes in ECG, heart rate, blood pressure, or sensory block.

Ten minutes later, a loading dose of 8 mL of the same mixture was given through the epidural catheter. After 20 minutes, a bilateral sensory block (to ice) to the T10 dermatome level was achieved. Patient-controlled epidural analgesia (PCEA) was started using an infusion of ropivacaine 0.1% with sufentanil 1 *μ*g/mL 5 mL/h with a patient-controlled bolus dose of 5 mL with a lockout interval of 30 minutes.

Labour was induced by amniotomy and an intravenous oxytocin infusion in increasing dosage, and the first stage progressed uneventfully.

At 9 cm dilatation, the patient experienced increasing pain which was managed by injection of 5 mL of lidocaine 1% through the epidural catheter, because of the pharmacologic profile of the fast onset and intensive blockade.

A healthy male neonate was spontaneously delivered 6.5 hours after starting the epidural. No hypotension or cardiac arrhythmias were recorded during this period, neither was a change in her heart block noted by the resident cardiologist.

The patient was continuously monitored for 24 hours postpartum on the obstetric high care unit; during this period no arrhythmias occurred.

## 3. Discussion

To our knowledge, this is the first case report in literature using epidural ropivacaine during labour in a patient with this gene mutation. For the diagnosis of BrS, the presence of a typical ECG pattern is mandatory. Our patient did not have this specific ECG pattern, although her ECG showed conduction disturbances. Because of her gene mutation and ECG abnormalities, she was advised to avoid the same medications as BrS patients.

A retrospective analysis of 104 pregnant Brugada patients with a total of 219 deliveries evaluated pregnancy outcomes. Three women, with an ICD inserted prior to pregnancy, had arrhythmia episodes recorded during pregnancy. Four other patients with aborted sudden cardiac death before pregnancy had no events during pregnancy. Of the 24 patients with syncope prior to pregnancy, only six experienced recurrent syncope during pregnancy [9]. However, a case report describes a patient, 12-week pregnant, presenting with a polymorphic ventricular tachycardia that failed to respond to routinely used drug therapy and defibrillation, as the first manifestation of BrS [10].

There are case reports describing the use of epidural bupivacaine infusions in male patients with BrS resulting in a Brugada-type ECG pattern [11], PVCs and ventricular fibrillation [12], and ventricular tachycardia and electrical storm in an undiagnosed Brugada patient with a SCN5A mutation [13]. There are however also cases with known BrS who had uneventful epidural analgesia [14, 15].

In contrast to spinal anesthesia, epidural analgesia requires a much higher volume of local anesthetic, therefore necessitating the use of the lowest effective concentration. Continuous epidural infusion with either ropivacaine or bupivacaine provides good labour pain relief, but use of ropivacaine resulted in higher plasma concentration in comparison to bupivacaine [16]. However, for a number of reasons, ropivacaine was considered the local anesthetic of choice. In our daily practice, we are used to the combination of ropivacaine 0.1% and sufentanil 1 *μ*g/mL, for our labour epidurals using PCEA. This mixture is prepared by our pharmacy, which reduces the risk of errors in medication. Bupivacaine seems to be more toxic than equivalent doses of ropivacaine with regard to the cardiovascular system. The PR interval, QRS duration, and QT interval (corrected for heart rate) are increased more when bupivacaine is given intravenously compared to intravenous ropivacaine, despite the mean plasma concentration of bupivacaine being lower than that of ropivacaine [17]. The cardiovascular changes, such as increased heart rate, decrease in stroke volume, and ejection fraction, are the same in both drugs. And finally, ropivacaine is presumably safer because it dissociates from the cardiac sodium channel more rapidly than bupivacaine does and thus produces a less pronounced inhibition of the cardiac sodium channel current [18].

In BrS patients, it is important that even the smallest ECG changes should be noticed and in case of changes epidural infusion must be discontinued [11]. For the perioperative monitoring, multilead ECG, preferably with ST trend analysis of the right cardiac leads, is advised [4, 19]. In this case, we did not perform but it is advisable in next cases.

As there is no clear advice in the literature on the duration of postoperative ECG monitoring in BrS patients, we continued ECG monitoring until 24 hours after removal of the epidural catheter. This decision was based on the fact that terminal half-life of ropivacaine after a 48-hour continuous epidural infusion is 7,4 hours [20]. Furthermore, Vernooy et al. [13] described the fact that the ventricular arrhythmias and the typical Brugada-like ECG disappear within 24 hours after discontinuing bupivacaine epidural infusion.

Good communication and cooperation between obstetricians, cardiologists, and anesthesiologists is mandatory. Furthermore, the patient should be informed of and involved in all decisions. One should avoid prolonged use of an epidural infusion and keep the concentration of the local anesthetic as low as possible, since the risk of provoking arrhythmia is related to the dose and duration of the medication used. There is always a risk of ventricular tachycardia or fibrillation, with potential disastrous consequences for mother and the unborn baby; a defibrillator should be on the ward to start advanced life support when necessary. Furthermore, a plan should be made for the management of hypotension and ventricular arrhythmias since the standard treatment is different from that in the ACLS plan. It is worth having isoprenaline available on the labour ward [4, 10].

With all precautions, we think that the use of ropivacaine for epidural analgesia during labour in patients considered as having SCN5A gene mutation with this phenotype is justified, although more experience and research are needed.
